# Gastroprotection of Suaveolol, Isolated from *Hyptis suaveolens*, against Ethanol-Induced Gastric Lesions in Wistar Rats: Role of Prostaglandins, Nitric Oxide and Sulfhydryls

**DOI:** 10.3390/molecules17088917

**Published:** 2012-07-26

**Authors:** Carlos Vera-Arzave, Leticia Cruz Antonio, Jesús Arrieta, Gerardo Cruz-Hernández, Antonio Magdiel Velázquez-Méndez, Adelfo Reyes-Ramírez, María Elena Sánchez-Mendoza

**Affiliations:** 1 Superior Medicine School, National Polytechnic Institute, Plan de San Luis y Díaz Mirón, Colonia Santo Tomás, Delegación Miguel Hidalgo, Mexico City, 11340, Mexico; Email: cvarzave@gmail.com (C.V.-A.); jearrval@yahoo.com.mx (J.A.); gerardocruz2282@hotmail.com (G.C.-H.); 2 Zaragoza Faculty of Higher Studies, Batalla del 5 de Mayo Esquina Fuerte de Loreto, Ejército de Oriente, Mexico City, 09230, Mexico; Email: letycruza@yahoo.com.mx (L.C.A.); adelfo@puma2.zaragoza.unam.mx (A.R.-R.); 3 Technological University of the Jungle, Entronque Toniná Carretera Ocosingo-Altamirano, Ocosingo, State of Chiapas, 29950, Mexico; Email: antoniomagdielv@yahoo.com

**Keywords:** *Hyptis suaveolens*, suaveolol, gastroprotection, medicinal plants

## Abstract

*Hyptis suaveolens* is a medicinal plant that is, according to traditional medicine, considered useful in the treatment of gastric ulcers. Although its gastroprotective activity was reported, the active compounds have not been identified. Therefore, the aim of the present study was to identify at least one active compound potentially responsible for the gastroprotective activity of *H. suaveolens* by using a bioassay guided study with an ethanol-induced gastric ulcer experimental model in rats. The results show that the hexane extract had protective activity (close to 70% when using doses between 10 and 100 mg/kg), and that the compound suaveolol, isolated from this extract, was one of the active gastroprotective agents. This is the first report about the gastroprotective activity of suaveolol. Rats treated with this compound at 3, 10, 30 and 100 mg/kg showed 12.6, 21.3, 39.6 and 70.2% gastroprotection respectively. The effect elicited by suaveolol (at 100 mg/kg) was attenuated by pretreatment with either *N*^G^-nitro-L-arginine methyl ester (70 mg/kg, i.p.), a nitric oxide (NO) synthase inhibitor, indomethacin (10 mg/kg, s.c.), a blocker of prostaglandin synthesis, or *N*-ethylmaleimide (10 mg/kg, s.c.), a blocker of sulfhydryl groups. This suggests that the gastroprotective mechanism of action of this compound involves NO, prostaglandins and sulfhydryl groups.

## 1. Introduction

Gastric ulcer, a major gastrointestinal disorder, results from an imbalance between offensive (gastric acid secretion) and defensive (gastric mucosal integrity) factors [1]. It is estimated that at some time in life, nearly 20% of all people suffer from peptic ulcers, caused by factors such as stress, diet, smoking, alcohol and certain types of drugs [2].

The drugs currently used in the treatment of gastric ulcers are antiacids, anticholinergics, proton pump inhibitors and H_2_-receptor antagonists. In the case of *H. pylori* infection, antibiotics are also used. However, the innumerable adverse effects caused by these allopathic medicines [3] evidence the need for safer and more effective antigastric ulcer agents. Metabolites derived from plants used in traditional medicine have provided an important basis for the discovery and development of modern therapeutic drugs [4].

*Hyptis* (Lamiaceae) is a genus comprising almost 400 species [5], some of which are used in traditional medicine in Latin America to heal wounds and treat gastrointestinal disorders, respiratory tract infections, colds, pain, fever, cramps, and skin diseases [6]. Previous phytochemical studies of this genus have shown that the constituents include hyptadienic acid, suaveolic acid, suaveolol, methyl suaveolate, β-sitosterol, oleanolic acid, ursolic acid, rosamarinic acid, dehydroabietinol, 3β-hydroxy-lup-12-en-28-oic acid, 3β-hydroxylup-20(29)-en-27-oic acid, and essential oils [7]. Among the species in the genus *Hyptis* is *H. suaveolens*, a fast-growing perennial herb found in dense clumps along roadsides, in over-grazed pastures and around stockyards in the tropics. Its branched, semi-woody stems can reach a height of 2 m. The plant gives off a characteristic minty smell when crushed. Originally native to tropical America, it is now considered a worldwide weed [8].

Although the essential oil obtained from *Hyptis spicigera* and the ethanolic and aqueous extracts of *Hyptis suaveolens* have shown gastroprotective activity in previous studies [9,10], there is no report of the compound responsible for this activity. Additionally, the aqueous extract of *H.suaveolens* obtained from leaves exhibits low acute or chronic toxicity [11,12]. In an attempt to find the active compound or compounds, we decided to evaluate the gastroprotective activity of *H. suaveolens* through a bioassay-guided study, using the absolute ethanol induced gastric ulcer experimental model in Wistar rats. Upon confirming gastroprotective activity and identifying the active compound, we explored the mechanism of action. Accordingly, we evaluated the role of endogenous NO, sulfhydryl groups and prostaglandins. The results were compared with the effect of carbenoxolone.

## 2. Results and Discussion

### 2.1. Bioassay-Guided Fractionation and Isolation of Suaveolol

Despite progress in diagnosis and treatment, peptic ulcer disease remains a common cause of hospitalization and surgery [13]. The current medicinal treatment of peptic ulcer is generally based on the inhibition of gastric acid secretion as well as on acid-independent therapy. In recent years, there has been growing interest in alternative therapies and the use of natural products, especially those derived from plants. In traditional medicine, several plants and herbs have been used to treat gastrointestinal disorders [14]. In this regard, extracts of hexane, dichloromethane and methanol from the aerial parts of *Hyptis suaveolens* were evaluated, finding that the hexane extract is the most active (Table 1). Interestingly, this effect was not dose dependent. This extract presented a maximum gastroprotective effect (70.0 ± 2.8%) at 10 mg/kg, with similar values obtained at 30 and 100 mg/kg (69.5 ± 6.9% and 69.7 ± 8.3%, respectively).

**Table 1:** Gastroprotective effect of *H. suaveolens* extracts on ethanol-induced ulceration in rats.

Treatment	Dose (mg/kg)	n	UI (mm^2^)	Gastroprotection (%)
Control	---	8	92.0 ± 7.0	---
Hexane extract	10	8	27.5 ± 2.6 *	70.0 ± 2.8
	30	8	28.1 ± 6.3 *	69.5 ± 6.9
	100	8	27.9 ± 7.6 *	69.7 ± 8.3
Dichloromethane extract	30	8	59.6 ± 2.5 *	35.2 ± 2.8
	100	8	40.3 ± 7.0 *	56.2 ± 7.7
Methanol extract	30	8	50.9 ± 6.8 *	44.7 ± 7.4
	100	8	42.7 ± 7.3 *	53.7 ± 8.5
Carbenoxolone	100	8	21.8 ± 3.9 *	72.0 ± 5.0

* *p* < 0.05 *vs.* control group; UI = Ulcer index.

**Figure 1:**
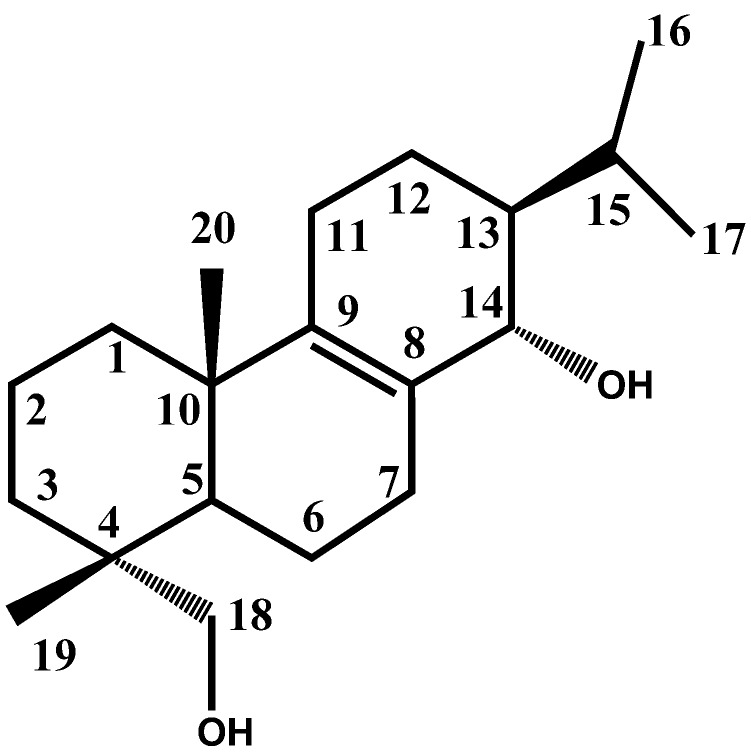
The structure of suaveolol.

Thus, 80 g of the hexane extract were separated over a silica gel column, giving four fractions (Table 2). Three of them had considerable activity, which suggests that *H. suaveolens* contains more than one active compound. F1 was found to be the most active, but the amount obtained of this fraction and F3 were minimal, and the compounds obtained when F1 was chromatographed on a silica gel column were unstable. Therefore, the decision was made to work with F2 in the present study. Accordingly, 35 g of F2 were separated on a silica gel column. Elution was performed with hexane and hexane/EtOAc mixtures, obtaining 50 fractions of 20 mL each. Fractions 45–50 (hexane/EtOAc, 8:2) yielded 2.5 g (7.1%) of a white solid (m.p. 182–183 °C). This solid was identified as suaveolol, an abietane diterpene (Figure 1), after a comparison between its spectral data (^1^H and ^13^C-NMR) and that in the literature [6]. Although suaveolol was previously isolated from *H. suaveolens*, only its anti-inflammatory activity has been reported [6]. In recent years, some reports have been published on the gastroprotective activity of diterpenes, as well as that of its derivatives belonging to different structural skeletons [15,16]. In the case of abietane diterpenes, some compounds showed greater gastroprotective effect than lansoprazole [17].

**Table 2:** Gastroprotective effect of the fractions of hexane extract on ethanol-induced ulceration in rats.

Treatment	Dose (mg/kg)	n	UI (mm^2^)	Gastroprotection (%)
Control	---	8	62.2 ± 4.2	---
F1	100	8	2.8 ± 1.2 *	95.5 ± 1.9
F2	100	8	22.3 ± 5.1 *	64.3 ± 8.3
F3	100	8	22.4 ± 5.0 *	63.9 ± 8.1
F4	100	8	43.0 ± 2.4	30.8 ± 3.8

* *p* < 0.05 *vs.* control group; UI = Ulcer index.

**Figure 2:**
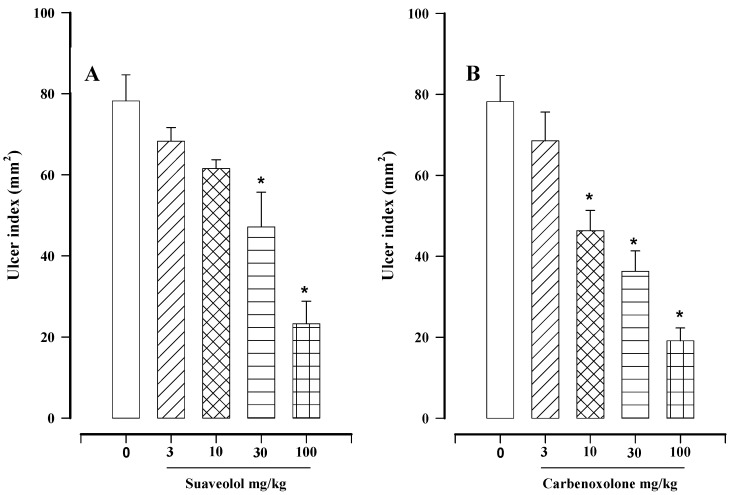
Effect of different doses of (**A**) suaveolol (3–100 mg/kg) and (**B**) carbenoxolone (3–100 mg/kg) on gastric lesions induced in rats by absolute ethanol. Bars represent the mean ± SEM, n = 8. *p < 0.05 *vs.* the respective control; Dunn’s multiple comparison test after the Kruskal-Wallis test.

In the current study, part of the gastroprotective activity of *H. suaveolens* was thus attributed to suaveolol, representing the first report of such activity by this compound. Suaveolol presented a gastroprotective effect (Figure 2A) similar to F2 (Table 2). Although there are apparently other compounds in *H. suaveolens*, apart from suaveolol, that contribute to its gastroprotective effect, they were not considered in this study because of the scarce quantity.

We found that suaveolol produced a dose dependent gastroprotective effect (Figure 2A), a condition not observed with the hexane extract (Table 1) from which suaveolol was obtained. This can be explained by the fact that the extract has more than one active compound, as shown by the fact three active fractions were obtained*.* The maximum gastroprotective effect of suaveolol was reached at a dose of 100 mg/kg (70.2 ± 7.1%), which was compared with the maximal gastroprotective effect induced by carbenoxolone (75.5 ± 5.5%), also at a dose of 100 mg/kg (Figure 2B). 

Although the mechanism of action of diterpenes has not been well established, these compounds seem to protect the gastric mucosa mainly through mechanisms that enhance the defensive factors of the stomach [18]. Therefore, in the present study we investigated the mechanism of action of suaveolol in relation to some gastric mucosa protective factors, such as nitric oxide, prostagladins and sulfhydryl compounds. 

### 2.2. Effect of L-NAME, Indomethacin*,* and NEM on the Gastroprotective Effect

Several studies have demonstrated the importance of endogenous NO in the protection of gastric mucosa. NO formed by endothelial NOS plays an important role in the modulation of gastric mucosal integrity by interacting with sensory neuropeptides and endogenous prostaglandins [19]. Considering that little information exists about the role of NO in the gastroprotection induced by abietane diterpenes, we decided to investigate the participation of NO in the mechanism of action of suaveolol, and for this purpose we used L-NAME (nitric oxide synthase inhibitor).

In the group treated with 70 mg/kg of L-NAME plus 100 mg/kg of suaveolol, the ulcer index was 128.3 ± 5.4 mm^2^ (Table 3), and this value was greater than that obtained with saline solution (90.9 ± 10.2 mm^2^). Thus, in the presence of L-NAME the gastroprotective effect of suaveolol was inhibited, indicating that NO is implicated in the mechanism of action. This result suggests that NO could be important in the gastroprotection of abietane compounds. The result obtained with the reference drug carbenoxolone (Table 3) was the same as that reported in the literature [4].

Gastric mucosal integrity requires the continuous generation of prostaglandin E_2_ (PGE_2_) and prostacyclin (PGI_2_). Inhibition of the synthesis of these molecules results in the reduction of blood flow and gastric mucosal damage [20]. Additionally, the gastroprotective activity of diterpenes and their derivatives has been explained by mechanisms that include stimulation of prostaglandin synthesis [17,18]. Therefore, in order to determine whether prostaglandins are involved in the mechanism of action of suaveolol, the animals were pretreated with indomethacin (10 mg/kg). 

**Table 3:** Effect of suaveolol and carbenoxolone on gastric lesions induced by ethanol in rats pretreated with L-NAME (70 mg/kg).

Treatment	Dose (mg/kg)	n	UI (mm^2^)
Control	---	10	90.9 ± 10.2
L-NAME	---	10	130.2 ± 7.7
L-NAME + suaveolol	100	10	128.3 ± 5.4
Suaveolol	100	10	19.1 ± 4.3 *
L-NAME + carbenoxolone	30	10	78.4 ± 9.4
Carbenoxolone	30	10	19.1 ± 3.1 *

* *p* < 0.05 *vs.* the respective control; UI = Ulcer index.

Indomethacin inhibited the gastroprotective action elicited by suaveolol (127.7 ± 9.2 mm^2^), evidenced by the fact that its ulcer index value was greater than that of the respective control using the vehicle (78.9 ± 6.3 mm^2^). Hence, prostaglandins also participate in the mechanism of action of suaveolol (Table 4), thus confirming the involvement of prostaglandins in the gastroprotection of the abietane diterpenes. Similarly, the gastroprotective effect of carbenoxolone was attenuated (77.3 ± 7.5 mm^2^) by pretreatment with indomethacin (Table 4), also indicating a role played by prostaglandins.

**Table 4:** Effect of suaveolol and carbenoxolone on gastric lesions induced by ethanol in rats pretreated with indomethacin (10 mg/kg).

Treatment	Dose (mg/kg)	n	UI (mm^2^)
Control	---	10	78.9 ± 6.3
Indomethacin	---	10	123.1 ± 4.8
Indomethacin + suaveolol	100	10	127.7 ± 9.2
Suaveolol	100	10	21.8 ± 6.1 *
Indomethacin + carbenoxolone	30	10	77.3 ± 7.5
Carbenoxolone	30	10	27.4 ± 5.5 *

* *p* < 0.05 *vs.* the respective control; UI = Ulcer index.

It is well known that reduced glutathione GSH protects the gastric mucosa submitted to an ulcerative challenge. It has been demonstrated that the development of ethanol induced gastric damage is accompanied by a decrease in mucosal sulfhydryl compounds, due to the fact that these compounds are neutralized when they bind to the free radicals produced with tissue injury by noxious agents [21]. Aiming to investigate the possible role of endogenous sulfhydryl compounds in the gastroprotective effect promoted by suaveolol, the animals were pretreated with NEM, a blocker of sulfhydryl compounds. This pretreatment produced a reduction in the gastroprotection exerted on ethanol-induced gastric lesions by the administration of suaveolol, evidenced by an increase in the value (176.5 ± 10.7 mm^2^) of the ulcer index (Table 5). A significant decrease in gastric GSH following ethanol administration indicated massive generation of free radicals [22]. In this context, the antiulcerogenic activity of suaveolol (100 mg/kg) may depend on mucosal GSH levels. Accordingly, it is likely that an increase of endogenous sulfhydryl compounds plays an important role in the gastroprotective properties of suaveolol, as is the case with other diterpenes, including the abietanes [15,17]. Regarding carbenoxolone, the results obtained with NEM are in agreement with the literature [4]. 

**Table 5:** Effect of suaveolol and carbenoxolone on gastric lesions induced by ethanol in rats pretreated with NEM (10 mg/kg).

Treatment	Dose (mg/kg)	n	UI (mm^2^)
Control	---	10	78.9 ± 6.3
NEM	---	10	81.2 ± 6.5
NEM + suaveolol	100	10	176.5 ± 10.7
Suaveolol	100	10	21.8 ± 6.1 *
NEM + carbenoxolone	30	10	70.8 ± 9.1
Carbenoxolone	30	10	27.4 ± 5.5 *

* *p* < 0.05 *vs.* the respective control; UI = Ulcer index.

## 3. Experimental

### 3.1. General Procedures

The ^1^H and ^13^C-NMR spectra were recorded in a CDCl_3_ solution on a VARIAN VXR-3005 spectrometer (Palo Alto, CA, USA), working at 400 and 100 MHz, respectively.

### 3.2. Plant Material

The leaves of *Hyptis suaveolens* were collected in the Municipality of Copainala, the State of Chiapas, Mexico during August of 2009. The plant was identified and registered by Francisco Hernández Najarro working in the Flora Department of the Chip Herbarium, which is part of the Botanical Garden of the Secretary of Environmental Protection, Housing and Natural History of the State of Chiapas, Mexico. A specimen of the original collection can be found with the voucher number 27939.

### 3.3. Extraction and Preliminary Fraction

The leaves of *H. suaveolens* were dried at room temperature (22 ± 2 °C) in the shade. After grinding 3 kg of leaves, compounds were successively extracted by maceration at room temperature (22 ± 2 °C) for 3 days, first with hexane (28 L × 3), then dichloromethane (28 L × 3) and finally methanol (28 L × 3). Evaporation of the solvents in vacuum yielded 77, 72, and 170 g of syrupy residues, respectively. The hexane extract obtained from the leaves of *H. suaveolens* showed the most active gastroprotective effect (Table 1). Thus 70 g of this extract were subjected to percolation over a silica gel column (particle size: 0.063–0.200 mm, 500 g) by using a step gradient of hexane (1.7 L, F1) hexane/EtOAc (9:1, 1.5 L, F2), hexane/EtOAc (7:3, 1.5 L, F3) and hexane/EtOAc (1:1, 1.5 L, F4). 10 g of the most active fraction (F1), were chromatographed on a silica gel column (200 g). However, the compounds obtained were unstable. Therefore, it was decided to work with fraction 2 (F2). 29 g of fraction 2 (F2) were chromatographed on a silica gel column (290 g) by using a step gradient of hexane, hexane/EtOAc and EtOAc. From this procedure, we obtained 50 fractions of 20 mL each. Fractions 15 to 25 (Hexane/EtOAc, 9:1) yielded a white solid (2.5 g, m.p. 182–83 °C), which was identified as suaveolol by comparing its spectral data (^1^H and ^13^C-NMR) with that of the literature [6].

### 3.4. Phytochemical Data: Suaveolol *(13*β*-abiet-8-ene-14*α*, 18-diol)*

^1^H-NMR (CDCl_3_): δ = 0.76 (3H, s, H19), 0.81 (3H, d, *J* = 8 Hz, H16), 0.95 (3H, d, *J* = 8 Hz, H17), 1.00 (3H, s, H20), 1.04 (1H, m, H1b), 1.09 (1H, m, H6b), 1.20 (1H, m, H13), 1.25 (1H, m, H3b), 1.40 (1H, m, H3a), 1.47 (1H, m, H12b or H5), 1.50 (1H, m, H5 or H12b), 1.56 (1H, m, H2b), 1.62 (1H, m, H2a), 1.65 (1H, m, H12a), 1.68 (1H, m, H6a), 1.72 (1H, m, H1a), 1.83 (1H, m, H7b or H11b), 1.88 (1H, m, H11b or H7b), 1.97 (1H, m, H15), 2.04 (1H, m, H7a), 2.20 (1H, s br, OH), 2.42 ( 1H, m, H11a), 2.68 (1H, s br, OH), 3.03 (1H, d, *J* = 12 Hz, H18b), 3.45 (1H, d, *J* = 12 Hz, H18a), 3.8 (1H, d, *J* = 8 Hz, H14). ^13^C-NMR (CDCl_3_): δ = 17.21 (C16), 17.74 (C19), 18.35 (C12 or C2), 18.40 (C2 or C12), 19.67 (C20), 21.46 (C17), 21.82 (C6), 24.52 (C7), 27.24 (C15), 28.05 (C11), 34.90 (C3), 35.85 (C1), 37.75 (C10 or C4), 37.78 (C4 or C10), 44.19 (C5), 48.98 (C13), 71.40 (C18), 73.38 (C14), 128.70 (C8), 142.82 (C9). These data match those in the literature [6].

### 3.5. Animals

All the experiments were performed with male Wistar rats, weighing 180–220 g, obtained from the animal house of the Universidad Autónoma Metropolitana Campus Xochimilco, in Mexico City, Mexico. Procedures involving animals and their care were conducted in conformity with the Mexican Official Norm for Animal Care and Handling (NOM-062-ZOO-1999), and in compliance with international rules on care and use of laboratory animals. Unless otherwise specified, the rats were placed in single cages with wire-net floors and deprived of food 24 h before experimentation. Animals were allowed free access to tap water throughout the experimental procedures. All experiments were carried out using 8–10 animals per group.

### 3.6. Drugs and Dosage

Carbenoxolone (Sigma-Aldrich Co. St. Louis, MO, USA) was used as the gastroprotective reference drug. This drug was prepared freshly for each use, suspended in 0.5% Tween 80 and administered by the intragastric route. Control rats received the vehicle (0.5% Tween 80) in the same volume (0.5 mL/100 g) and by the same route. *N*^G^-nitro-L-arginine methyl ester (L-NAME), *N*-ethylmaleimide (NEM) and indomethacin (IND) were purchased from Sigma Chemical Co. (St. Louis, MO, USA).

### 3.7. Acute Gastric Ulcer Induced by Absolute Ethanol

A gastric ulcer was induced by administering absolute ethanol orally (1 mL) [4]. The extracts or drugs were administered to different groups 30 min before ethanol administration. Two hours after ethanol administration, the animals were sacrificed in a CO_2_ chamber. The stomach and duodenum were dissected, inflated with formalin (10 mL), and then placed in 2% formalin for 5 min to fix both the inner and outer layers. The duodenum was opened along its anti-mesenteric side and the stomach along the greater curvature. The damaged area (mm^2^) was measured under a dissection microscope (×10) with an ocular micrometer. The ulcer index was calculated as the sum of all the lesions (area in mm^2^) in the stomach of each animal. Gastroprotection (%) was calculated according to: % gastroprotection = (UIC − UIT) × 100/UIC, where UIC is the ulcer index in control and UIT is the test animal index [4].

### 3.8. Ethanol-Induced Gastric Mucosal Lesions in L-NAME Pretreated Rats

To investigate the involvement of endogenous NO in the gastroprotective effects of the compounds, L-NAME (70 mg/kg dissolved in saline solution) was intraperitoneally injected into 3 experimental groups 30 min before the administration of either the vehicle, suaveolol (100 mg/kg) or carbenoxolone (100 mg/kg) by the oral route [4]. Absolute ethanol was given to each rat in these groups 30 min later, and the animals were sacrificed 2 h after the administration of ethanol to measure the ulcer index. Two control groups (L-NAME-treated and non-L-NAME-treated) were included in this evaluation. 

### 3.9. Ethanol-Induced Gastric Mucosal Lesions in Indomethacin Pretreated Rats

To investigate the involvement of endogenous prostaglandins in the gastroprotective effect of the compounds, a control group received a subcutaneous injection of 5 mM NaHCO_3_ in saline solution and another group an injection of indomethacin (10 mg/kg dissolved in NaHCO_3_ at 5 mM) by the same route [4]. After 75 min, the animals in each of these two groups received one of three oral treatments (saline solution, 100 mg/kg suaveolol or 100 mg/kg carbenoxolone). Absolute ethanol was given to each rat 30 min after suaveolol or carbenoxolone administration and the rats were sacrificed 2 h later in a CO_2_ chamber. The stomachs were subsequently removed to measure the ulcer index, as aforementioned.

### 3.10. Ethanol-Induced Gastric Mucosal Lesions in NEM Pretreated Rats

To investigate the involvement of the endogenous sulfhydryls in the protective effects of suaveolol and carbenoxolone, NEM (dissolved in saline solution) was subcutaneously injected (10 mg/kg) in 3 groups of animals 30 min before the oral administration of either the vehicle, suaveolol (100 mg/kg) or carbenoxolone (100 mg/kg) [4]. Absolute ethanol was given to each rat 30 min later and rats were sacrificed 2 h after the administration of ethanol to measure the intensity of the gastric ulcer. Two control groups (NEM-treated and non-NEM-treated) were included in this experiment.

### 3.11. Statistics

Data are presented as the mean ± SEM from 8–10 rats per group. Statistical significance between the treatments was evaluated by the Kruskal-Wallis test, followed by Dunn’s multiple comparison tests, with *p*< 0.05 considered as significant.

## 4. Conclusions

The current study demonstrates the effectiveness of *Hyptis suaveolens* in the treatment of gastric ulcer. Suaveolol was identiﬁed as the main active gastroprotective agent in this traditional medicinal plant. The mechanism of the gastroprotective action shown by suaveolol is related to endogenous NO, prostaglandins and sulfhydryl groups.
